# An Accessible Alternative to Melarsomine: “Moxi-Doxy” for Treatment of Adult Heartworm Infection in Dogs

**DOI:** 10.3389/fvets.2021.702018

**Published:** 2021-07-27

**Authors:** Linda S. Jacobson, Brian A. DiGangi

**Affiliations:** ^1^Shelter Medicine Advancement, Toronto Humane Society, Toronto, ON, Canada; ^2^Shelter and Veterinary Services, American Society for the Prevention of Cruelty to Animals, New York, NY, United States

**Keywords:** heartworm, *Dirofilaria immitis*, slow kill, moxidectin, doxycycline, accessible veterinary care, adulticide treatment, melarsomine

## Abstract

Canine heartworm infection, caused by the filarial parasite *Dirofilaria immitis*, represents a serious and expanding animal welfare concern that is expected to increase due to the effects of climate change and the COVID-19 pandemic. A body of evidence has emerged to support the use of a non-arsenical adulticide treatment protocol, using moxidectin and doxycycline to kill adult heartworms over a prolonged period. While a three-dose protocol using the arsenical drug melarsomine is currently the safest and most effective treatment for heartworm infection, this drug is not available in some countries and is inaccessible for many owners and animal shelters. Moxidectin-doxycycline (moxi-doxy) provides a viable alternative to no treatment at all, in cases where arsenical treatment is not possible. Based on current evidence, the most effective non-arsenical treatment regimen is doxycycline 10 mg/kg PO q 12 or 24 h for 28 days, combined with topical moxidectin at label dose. Moxidectin is repeated monthly until no antigen detected (NAD) status is confirmed. Sustained release injectable moxidectin, in combination with doxycycline, may provide an alternative in remote regions or in settings where significant compliance or accessibility concerns exist, but more studies are needed. In moxi-doxy protocols, doxycycline should be repeated annually until NAD. This review summarizes the safety and efficacy of moxi-doxy, addresses controversies surrounding this treatment approach, and provides detailed recommendations for treatment regimens and post-treatment testing.

## Introduction

Current guidelines for the treatment of adult heartworm (*Dirofilaria immitis*) infections in dogs recommend 3 doses of the arsenical drug, melarsomine dihydrochloride, accompanied by doxycycline and a macrocyclic lactone (ML) (1–3). This is considered to be the safest, most rapid and most efficacious treatment protocol. It is the only protocol recommended for treatment of severe heartworm disease (HWD) unless the disease is so severe that surgical intervention is warranted or life-threatening adverse effects to melarsomine and/or its parasiticidal results are anticipated (2, 4).

Although no longer considered best practice, melarsomine is labeled for 2 doses, 24 h apart, in dogs with asymptomatic, mild or moderate HWD (2). This protocol is commonly used in animal shelters, to reduce cost and length of stay (1, 5). The 2-dose protocol (with no adjunctive treatment) killed ~90% of adult worms, compared with 99% after 3 doses (6).

Despite the medical and scientific soundness of this approach, melarsomine treatment remains unattainable for many affected dogs and their caregivers (including many animal shelters) (5, 7–10). Due to increasing recognition of this problem, expanding pharmaceutical options, and a rapidly-growing scientific evidence base, practitioners have experimented with non-arsenical treatment protocols with little professional industry guidance.

The purpose of this review is to review the current literature on the use of non-arsenical protocols for HWD management, within an accessible care context and with an emphasis on the combination of moxidectin and doxycycline (moxi-doxy); and offer an evidence-based and informed “least harm” approach for practitioners to consider when necessary.

## Is Perfect the Enemy of Good When it Comes to Heartworm Treatment?

*Dirofilaria immitis* is a significant emerging infection globally (10). The incidence is reported to be increasing in some endemic areas and spreading to regions in which it was not previously identified (11–13). This trend is expected to continue, exacerbated by expanding ranges resulting from climate change (13) and by the economic consequences of both climate change and the COVID-19 pandemic (14–16). These forces will deepen existing societal and economic inequities, which impact the social determinants of health for animals (7) as they do for humans (17). For these reasons, there is a growing imperative to specifically address the need for accessible and affordable treatment for heartworm infection (HWI), alongside efforts to more broadly remove barriers to veterinary care – a problem that affects over 27% of pet-owning households in the United States and has been called “the most significant animal welfare crisis affecting owned pets” (7). Reasons for pet owners' inability to access veterinary care—including heartworm prevention and treatment—include cost, not knowing where to seek care, lack of transportation, and lack of equipment such as carriers and leashes (7).

**Figure 1:**
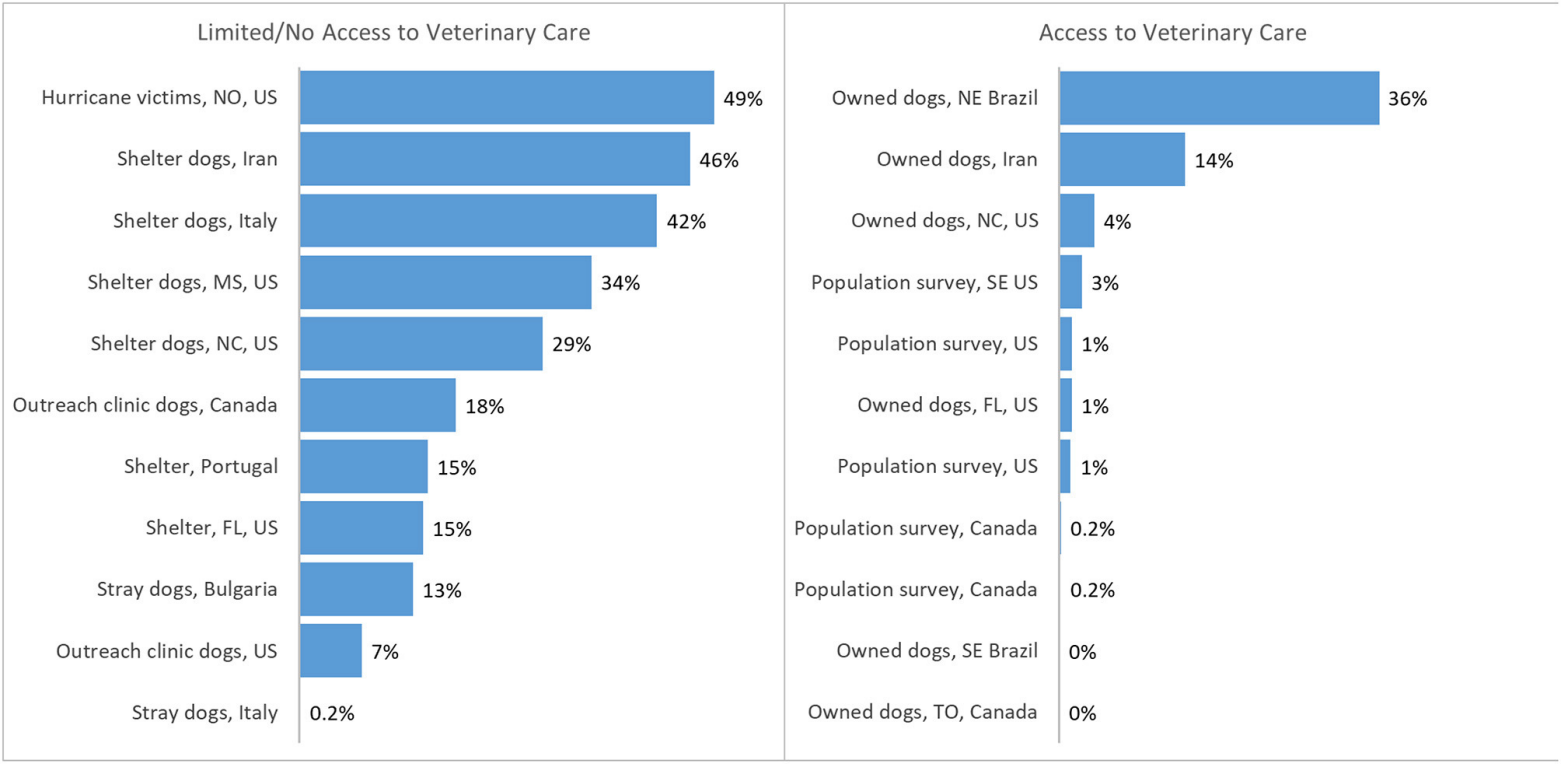
Prevalence or incidence of heartworm infection reported in populations of dogs with access (left) or with limited/no access (right) to veterinary care (20–30, 34–39). Population surveys = data from reference laboratories ± veterinary clinics. Portugal data averaged between 3 sites and over 3 years. TO, Toronto, Canada; US, United States; FL, Florida; NE, northeast; SE, southeast; NC, North Carolina; MS, Mississippi; NO, New Orleans.

Heartworm has been described as a socioeconomic disease (18). Lower household income has been associated with higher prevalence, most likely because families were unable to purchase heartworm preventives (19). In most surveys, prevalence is markedly greater in dogs with limited or no access to veterinary care than owned dogs likely to have been tested at veterinary visits (12, 20–30) (Figure 1). Owners of heartworm-positive dogs are frequently unable to afford or otherwise access melarsomine treatment (8, 9, 18, 31, 32), which is expensive and requires multiple veterinary visits. The cost of treatment was reported by shelters to be the most important challenge for treating HWI (5). Concerns about pain at the injection site, drug toxicity, and the requirement for prolonged and strict exercise restriction are additional reasons that owners may elect alternative treatment protocols, even if melarsomine treatment is available to them.

Heartworm is a significant and growing concern for animal shelters in the US (1). When creating treatment protocols for affected populations of dogs in shelters, individual animal care must be balanced with limited resources, and division of those resources (including personnel to treat) to benefit as many animals as possible. The Association of Shelter Veterinarians' Position Statement on Heartworm Management acknowledges this balance, by encouraging shelters to treat affected dogs if possible, but to also ensure that “resources diverted toward heartworm management do not compromise the care of other shelter animals” (1). This Position Statement also provides background information, with a comprehensive tabulated comparison of a variety of arsenical and non-arsenical adulticidal protocols (1). The long duration of treatment coupled with the detrimental effects of increased length of stay in the shelter system, the cost of care throughout treatment, and the expense of melarsomine itself make this disease particularly challenging in shelter populations (40).

Even when resources are available, the lack of availability of melarsomine in some countries and the occurrence of multiple supply shortages in the past decade can leave large populations of dogs reliant on alternative treatments. Thus, while melarsomine treatment is acknowledged as best practice, safe and effective non-arsenical therapies are necessary to ensure that viable treatment options are available to all dogs with HWI.

In light of these concerns, many veterinarians have applied a “least harm principle” to heartworm management. This principle asserts that in the face of a situation in which both options may have negative consequences, the decision-maker should choose that which results in the least harm possible (41–43). In the case of treating a heartworm positive dog for which a melarsomine-based protocol is not feasible, the practitioner is faced with the option of not treating the dog or employing a non-arsenical approach. The authors contend that there is now sufficient evidence to support the use of non-arsenical treatment in many of these scenarios. Furthermore, such approaches can break the cycle of transmission, thereby benefiting not only the individual patient and owner but the larger population of susceptible animals.

## Non-Arsenical Adulticide Treatment Protocols

Non-arsenical adulticide protocols that utilize a prolonged course of an ML have historically been labeled “slow-kill” or “soft-kill” (44, 45). Although the term “slow-kill” is still frequently used, it can refer to a variety of protocols with wide variations in evidence regarding the duration of use, safety, and efficacy. In the authors' experience, this term also often implies a safer option to many pet owners and carries a negative connotation with others. For all these reasons, we suggest that these terms should be avoided, and more objective and descriptive terminology used in their place.

Non-arsenical treatment is not recommended as a first-line treatment by the American Heartworm Society (AHS) and the Companion Animal Parasite Council (CAPC) (2, 46) and is not addressed in the European (ESCCAP) guidelines (3). The AHS Guidelines state that, in cases where melarsomine treatment is not possible or is contraindicated, treatment with doxycycline and an ML can be considered as a “salvage procedure” (2). This term generally refers to conditions that are refractory to other available treatment, or where the patient is unable to tolerate other treatments (47); thus excluding indications for use that lie outside of the individual patient's clinical condition, such as financial considerations, access to follow-up care and population-level impact. We contend that non-arsenical treatment is a reasonable alternative in specific circumstances and should be presented as such.

Outside of accessibility considerations, non-arsenical treatment is indicated if there is a history of a life-threatening adverse reaction to melarsomine; comorbidity conferring a guarded or grave prognosis; comorbidity making deep epaxial injection impracticable or during stabilization of severe heartworm-induced cor pulmonale (48). Despite recommendations against use in professional practice guidelines, non-arsenical protocols are widely used in certain circumstances and settings (5, 8–10, 32, 40, 49–51).

While numerous concerns exist regarding non-arsenical protocols, multiple studies have now demonstrated the safety and efficacy of moxi-doxy protocols (Table 1). These protocols tend to be used with variations, leading to a lack of uniformity in approach, and uncertainty on the part of practitioners and shelters faced with their implementation. A more standardized, evidence-based, approach is necessary to support practitioners utilizing these therapies.

**Table 1:** Efficacy of moxidectin ± doxycycline for heartworm adulticide treatment in dogs.

**References**	***n***	**Setting**	**Treatment protocol**	**Efficacy^**a**^**	**Time point**
Paterson et al. (9)	15	NI	Moxidectin (2.5 mg/kg) + imidacloprid (10 mg/kg) applied topically q 30 d ×9 m; restarted at 15 m. Doxycycline 10 mg/kg q 12 h PO ×28 days.	7%	3 m^b^
				13%	6 m
				73%	12 m
				92%	18 m
	15	NI	Melarsomine 2.5 mg/kg IM, repeated after 24 h	57%	3 m
				79%	6 m
				86%	12 m
				100%	18 m
Genchi et al. (52)	14	NI	Moxidectin (2.5 mg/kg) + imidacloprid (10 mg/kg) applied topically q 30 d ×9 m; doxycycline 10 mg/kg PO q 12 h ×30 d	93%	9 m
				100%	12 m
				100%	18 m
	6	NI	Melarsomine 2.5 mg/kg IM at enrollment, repeated twice, 24 h apart, after 1 m	17%	3 m
				100%	6 m
				83%	12 m
				100%	18 m
Bendas et al. (49)	22	NI	Moxidectin (2.5 mg/kg) + imidacloprid (10 mg/kg) applied topically q 30 d until the study definition of negative was met; doxycycline 10 mg/kg q 12 h PO ×30 d, repeated q 6 m	67%	6 m
				95%	12 m
				100%	18 m
Ames et al. (48)	22	NI	Moxidectin (2.5 mg/kg) + imidacloprid (10 mg/kg) applied topically q 15 d for 90 d, then q 30 d until negative. Doxycycline 12–16 mg/kg PO q 24 h ×15 d; additional courses (doxycycline or minocycline) at clinicians' discretion.	32%	6 m
				86%	12 m
				95%	18 m
				95%	24 m
Savadelis et al. (53)	8	EI	Moxidectin (2.5 mg/kg) + imidacloprid (10 mg/kg) applied topically q 30 d ×10 m; doxycycline 10 mg/kg PO q 12 h ×30 d	96%	10 m
McCall et al. (54)	5	EI	Moxidectin 0.17 mg/kg subcutaneously, once	25%	6 m
	5		Moxidectin 0.17 mg/kg subcutaneously, q 6 m ×3	5%	18 m
Alberigi et al. (55)	20	NI	Moxidectin 0.5 mg/kg subcutaneously q 6 m; doxycycline 10 mg/kg PO q 12 h ×30 days, repeated q 6 mths	55%	6 m
				90%	12 m
				100%	27 m

*Topical moxidectin = Advantage Multi/Advocate®. Subcutaneous moxidectin = ProHeart® 6 precursor (54); ProHeart® 12 (55). Time point, months from initiation of treatment. NI, natural infection; EI, experimental infection*.

a *Defined here as % worms absent or non-viable at necropsy, for experimental infections; % negative at first antigen test, for natural infections (other than for Paterson et al., where NAD was defined as 2 negative antigen tests and the timing for the first NAD was not provided)*.

b *Calculated from Figure 1 in that report*.

## Efficacy of Non-Arsenical Adulticide Protocols

Studies showing the efficacy of moxi-doxy are summarized in Table 1, with selected studies of ivermectin (IVM) in Table 2 and melarsomine in Table 3, for comparison. This review will focus on moxidectin and IVM, as other MLs such as milbemycin and selamectin have poor efficacy for the elimination of adult worms (45).

**Table 2:** Efficacy of ivermectin ± doxycycline for heartworm adulticide treatment in dogs.

**References**	***n***	**Setting**	**Treatment protocol**	**Efficacy^**a**^**	**Time point**
Bazzocchi et al. (56)	5	EI	Ivermectin 6 μg/kg weekly for 34 weeks	20%	9 m
	5		Ivermectin 6 μg/kg PO q 7 d ×34 weeks; doxycycline 10 mg/kg PO q 12 h ×6 weeks then 2 weeks out of 4, until 34 weeks	78%	9 m
Grandi et al. (57)	11	NI	Ivermectin^b^ 6 μg/kg PO q 15 d ×6 m; doxycycline 10 mg/kg q 24 h ×30 d	18%	3 m
				45%	6 m
				73%	10 m
McCall et al. (58)	5	EI	Ivermectin^c^ 6 μg/kg PO q 7 d ×9 m	20%	9 m
	5		Ivermectin 6 μg/kg PO q 7 d ×36 w; doxycycline 10 mg/kg q 24 h during weeks 1–6, 10–11, 16–17, 22–25, 28–33	78%	9 m
	5		Melarsomine 2.5 mg/kg IM once at week 24, two doses 24 h apart at week 28; ivermectin 6 μg/kg PO q 7 d ×36 w; doxycycline 10 mg/kg q 24 h during weeks 1–6, 10–11, 16–17, 22–25, 28–33	93%	9 m
	5		Melarsomine 2.5 mg/kg IM once at week 24, two doses 24 h apart at week 28	100%	9 m

*Time point, number of months from initiation of treatment. EI, experimental infection; NI, natural infection*.

a *% worms absent or non-viable at necropsy, for experimental infections, % with negative antigen test, for natural infections*.

b *Cardotek Plus® with pyrantel pamoate*.

c *Heartgard® with pyrantel pamoate*.

**Table 3:** Efficacy of melarsomine for heartworm adulticide treatment in dogs.

**References**	***n***	**Setting**	**Treatment protocol**	**Efficacy^**a**^**	**Time point**
Keister et al. (6)	6	EI	Melarsomine 2.5 mg/kg IM once	52%	2 m
	9		Melarsomine 2.5 mg/kg IM, repeated after 24 h	91%	2 m
	6		Melarsomine 2.5 mg/kg IM once; then 30 days later 2 doses of 2.5 mg/kg IM, 24 h apart	99%	3 m
Miller et al. (59)	42	NI	Melarsomine 2.5 mg/kg IM once, repeated after 24 h	76%	4 m
	39	NI	Melarsomine 2.5 mg/kg IM once, repeated after 24 h; treatment course repeated after 4 m	90%	8 m

*Time point, number of months from initiation of treatment; EI, experimental infection; NI, natural infection*.

a *% worms absent or non-viable at necropsy for experimental infections, % with negative antigen test for natural infections*.

Measures of efficacy depend on study type. Necropsy studies utilize counts of adult worms retrieved, while most clinical studies utilize antigen test results, sometimes in conjunction with echocardiography. Necropsy studies provide the most reliable measure of efficacy.

### Efficacy of Ivermectin

A series of studies of the effects of IVM on different stages of *D. immitis* has been elegantly reviewed (45). Monthly IVM was highly effective against immature worms but required a longer duration of treatment to clear adult worms (45). The efficacy, at necropsy, for adult worms was 56% after 16 months (8-month-old worms) (60) and 95% after 29 months (7-month-old worms) (44). These studies used preventive dosage regimens, i.e., 6 μg/kg monthly.

The combination of IVM and doxycycline (IVM-doxy) demonstrated synergistic activity against adult worms compared with IVM alone (Table 2) (56, 58). Efficacy was 78% at 9 months (56), 73% at 10 months (57), and 78% at 9 months (58) after initiation of treatment. In these studies, IVM was used at the preventive dose of 6 μg/kg, but dosing frequency was increased to every 7 or 14 days (Table 2). Dogs with Class 3 HWD had higher worm burdens and poorer IVM-doxy adulticide efficacy, compared with Class 1 and Class 2 disease (61).

Ivermectin offers the advantage of being less expensive than moxidectin. However, despite improved adulticide efficacy when doxycycline is added, this combination is less effective than moxi-doxy and requires more frequent administration (Tables 1, 2). No published studies have directly compared IVM-doxy and moxi-doxy.

### Efficacy of Moxidectin

Several studies have now evaluated moxi-doxy for adulticidal treatment, with encouraging results (Table 1) (9, 31, 48, 49, 52, 53, 55). As yet, no randomized, controlled trial (RCT) has directly compared the safety and efficacy of the current AHS-recommended melarsomine-based protocol and moxi-doxy.

The greater adulticide efficacy of moxidectin compared with IVM (Tables 1, 2) may be due to its unique pharmacokinetics and pharmacodynamics, which result in sustained exposure of the worms (62). Compared with IVM, moxidectin is more lipophilic, with a larger volume of distribution, longer half-life and slower elimination (62–64). Serum concentrations remain high for at least 28 days after administration of topical moxidectin at label dose, with steady state being reached after four monthly treatments (65). This is in contrast to other ML heartworm preventives, which have shorter half-lives and rely on reach-back effects to eliminate microfilariae acquired during the previous month (45, 65).

Moxidectin is available as a heartworm preventive in topical, sustained release (SR) injectable and oral formulations (64, 66–68). The labeled moxidectin dosage regimens are:

- Topical moxidectin (Advantage Multi®, Elanco; IMOXI, Vetoquinol; 100 mg/mL imidacloprid, 25 mg/mL moxidectin) – 2.5 mg/kg moxidectin applied to the skin once a month.
- SR moxidectin (ProHeart® 6; 3.4 mg/mL when constituted) – 0.17 mg/kg SQ once every 6 months;
- SR moxidectin (ProHeart® 12; 10 mg/mL when constituted) – 0.5 mg/kg SQ once every 12 months;
- Oral moxidectin (Simparica Trio; relative ratios 12 mg sarolaner: 240 μg moxidectin: 50 mg pyrantel; tablet sizes vary) - 24 μg/kg moxidectin orally once a month.

Topical moxidectin is the only ML formulation labeled for use in microfilaremic dogs (69) and clears microfilariae rapidly and effectively (70). Other MLs are relatively safe for microfilaremic dogs, at the prescribed dose and under veterinary supervision, but efficacy and clearance times are variable (71).

#### Topical Moxidectin

Five topical moxi-doxy studies have been published. Salient features are provided here, with further details summarized in Table 1.

All five studies used 10% moxidectin + 2.5% imidacloprid (Advantage Multi® or Advocate®, Elanco). Three of the study protocols administered the product every 30 days for 9 or 10 months (9, 52, 53), with resumption of monthly treatments at 15 months in one (9). In one study, monthly administration was continued until the study definition for NAD was met (49). In another, topical moxidectin was administered every 15 days for 90 days and every 30 days thereafter until NAD (48).

Doxycycline was administered at the recommended (2) dosage of 10 mg/kg q 12 h for 28–30 days in four studies (9, 49, 52, 53) and at 12–16 mg/kg q 24 h for 15 days in one (48). The course was repeated after 6 months (49); at varying intervals (doxycycline or minocycline) based on clinician discretion (48), or either not specified or not repeated in the other 3 studies.

- One RCT has been published, comparing moxi-doxy with melarsomine in 30 naturally infected dogs (15 per group) (9). Melarsomine was administered as a 2-dose protocol without doxycycline. Decline in antigenemia and NAD status was achieved more rapidly for melarsomine-treated dogs. Seven percent of the dogs in the moxi-doxy group had NAD at 3 months, 13% at 6 months, 73% at 12 months, and 92% at 18 months. Two dogs in the moxi-doxy group remained antigenemic, at 12 months and 18 months respectively, and were treated with melarsomine.
- One other study directly compared moxi-doxy and 3-dose melarsomine in naturally infected dogs (52). The study was not randomized and the melarsomine group did not receive doxycycline. Fourteen dogs received moxi-doxy and 6 received melarsomine. All dogs in the melarsomine group had NAD status at 6 months after administration, with one testing transiently positive at 12 months. Thirteen of 14 dogs in the moxi-doxy group (93%) had NAD at 9 months, and all tested negative at 12, 18 and 24 months. Moxi-doxy was statistically non-inferior to melarsomine at 12, 18, and 24 months.
- Another study reported results of moxi-doxy treatment in 22 naturally infected dogs (49). All dogs had NAD at 18 months, with 64% testing negative at 6 months and 96% at 12 months. Dogs with lower microfilaria counts tended to test negative sooner than those with higher counts, suggesting that efficacy may be negatively associated with worm burden and/or worm age.
- A study of 22 naturally infected dogs reported NAD status in 27% at 6 months, 86% at 12 months and 95% at 18 months, with one treatment failure as evidenced by a positive antigen test result at day 701 (48).
- An experimental study used 16 experimentally infected dogs, 8 of which received moxi-doxy and 8 of which were untreated controls. Worm counts at necropsy were used to assess efficacy. The study reported 96% efficacy of moxi-doxy against adult worms after 10 months of treatment, with 5 dogs completely negative and 1–2 worms each recovered from the remaining three dogs (53). In untreated controls, adult worms were recovered from all dogs, with numbers ranging from 10 to 12 per dog.

In summary, topical moxidectin plus doxycycline has shown good efficacy across different study designs, dog populations and dosage regimens, with some dogs testing negative as early as 3–6 months after initiation of treatment and the majority reaching negative or NAD status by 10–18 months. A small number of treatment failures have been recorded.

#### Sustained Release Injectable Moxidectin

SR injectable moxidectin products (ProHeart® 6 and Proheart® 12, Zoetis) have been in use as heartworm preventives for many years (64, 68, 72) and have been widely adopted in Australia as well as marketed more recently in several Asian and Latin American countries. These products contain microspheres of moxidectin that are gradually released over a prolonged period of time, either 6 (ProHeart® 6) or 12 (ProHeart 12®) months depending on concentration and dose. SR formulations were designed to circumvent the poor compliance with monthly preventive administration that frequently occurs (29, 45, 73, 74).

The 6-month SR product was temporarily recalled in the US in 2004 due to concerns about severe anaphylactoid reactions within the first 48 h of treatment^1^. An extensive pharmacovigilance monitoring program and post-marketing surveillance showed allergic reactions of 1.26 per 10,000, with a similar rate (1.19) for non-allergic reactions (75). Recent studies of 12-month SR moxidectin demonstrated safety and efficacy as a preventive (64, 76). Reports of adverse events over an extended period were comparable to those reported for ivermectin/pyrantel (Heartgard® Plus, Boehringer Ingelheim) (64).

Two published studies have assessed the effect of SR moxidectin on adult heartworms (Table 1). Administration of 0.17 mg/kg of SR moxidectin alone (without doxycycline) had poor efficacy for clearing adult heartworms in experimental dogs; however, many worms were abnormal at necropsy (54). A recent study assessed the efficacy of SR moxidectin combined with doxycycline in 20 naturally infected dogs (55). Twelve-month SR moxidectin was given every 6 months (i.e., at twice the label frequency) and doxycycline was dosed at 10 mg/kg q 12 h for 30 days, repeated every 6 months. The injection frequency was selected to maintain higher serum concentrations. Efficacy was comparable to results from topical moxidectin, with 55% NAD at 6 months, 90% at 12 months and 100% at 27 months. In a related case report, a dog treated with this protocol was found to be pregnant before the second moxidectin injection and gave birth 1 week after the injection. Doxycycline was not repeated at the 6 month visit (77). She had NAD at 6 and 12 months, and there were no observed adverse effects for her or her puppies.

In a survey-based study examining veterinary practices at a clinic in Mississippi, United States, 6-month SR moxidectin was the most frequently prescribed non-arsenical protocol and was selected by clients who were unable to afford arsenical treatment (32). As this study was survey-based, no information about the success or outcomes of those cases was provided.

If supported by further studies, SR moxidectin would be a useful alternative to topical moxidectin for adulticide treatment in remote regions such as northern Canada, or in circumstances where a long-acting treatment is preferable due to accessibility and/or compliance issues.

#### Oral Moxidectin

To the authors' knowledge, oral moxidectin (Simparica Trio®, Zoetis) has not been evaluated for adulticidal efficacy.

### Moxi-Doxy: How Long Should Treatment Continue?

Depending on the study purpose and design, the duration of treatment has varied (Table 1). Findings indicate that worm death rates approaching those achieved with melarsomine-based protocols should not be expected prior to ~10 months of continuous therapy. Given the unpredictable time to NAD status in an individual dog, moxidectin should be continued until NAD status has been confirmed. This could be defined as one or two negative antigen tests, as discussed below. Doxycycline should be repeated annually for dogs still testing positive, as mentioned below.

## Doxycycline in Non-Arsenical Adulticide Protocols

Doxycycline has become a necessary component of heartworm treatment (2), because of its effects on the filarial endosymbiont bacteria, *Wolbachia* (78, 79). The addition of doxycycline markedly reduces the pulmonary pathology associated with dead and dying worms, reduces the risk of thromboembolism and disrupts heartworm development and transmission (33, 80–82). When given alone to dogs experimentally infected with third stage *D. immitis* larvae, doxycycline prevented the development of adult worms, with decreased effectiveness against older immature stages (83).

McCall et al. treated dogs experimentally infected with third-stage larvae (L3) with doxycycline for 1 month at 20 mg/kg/day and then verified the presence of adult worms at 8 months post-infection (83). When doxycycline was administered during the first month post-infection, no live worms were observed at necropsy, indicating 100% efficacy against infective larvae. When doxycycline was commenced 40 days post-infection, efficacy against developing worms was 98%. Finally, when treatment was initiated 65 days post-infection, efficacy against juvenile worms was 70%. Interestingly, none of the dogs that harbored live worms was microfilaremic.

The combination of doxycycline (20 mg/kg q 24 h ×30 d), IVM (6 μg/kg monthly) and melarsomine (2.5 mg/kg IM, followed by 2 injections 24 h apart 1 month later) resulted in dramatically reduced pulmonary perivascular inflammation and endothelial proliferation after treatment of HWI, compared with doxycycline alone or melarsomine alone (82). Doxycycline also accelerates adult worm clearance and improves adulticidal efficacy when used with MLs (56, 58, 84) (Table 2). Worms found at necropsy were abnormal on histology and electron microscopy, with marked alterations in embryogenesis (56) and it was suspected that most worms that persisted in dogs treated with IVM-doxy would not have survived (58).

### Dosage and Duration of Doxycycline

In the typical 3-dose melarsomine protocol, doxycycline is started at the time of diagnosis and continued for 28–30 days (2, 3). The dosage recommended by the AHS is 10 mg/kg PO q 12 h (2), while the ESCCAP Guidelines recommend 10 mg/kg PO q 24 h (3) and the CAPC Guidelines do not specify a dosage (46). Both 10 and 20 mg/kg/day have been used successfully in both arsenical and non-arsenical protocols, but no RCT has compared their parasitological efficacy. Tables 1, 2 detail dosages of doxycycline utilized in non-arsenical adulticide studies.

Doxycycline or minocycline, at either 5 mg/kg or 10 mg/kg q 12 h for 28 days, were compared in a randomized study of 32 microfilaremic dogs with HWI (85). The 3-dose melarsomine protocol was used with IVM. There was no significant difference in the rate of microfilarial clearance between the groups. Microfilariae in all 8 dogs treated with 10 mg/kg doxycycline q 12 h were negative for *Wolbachia* DNA by day 28. Microfilariae in 2/8 dogs treated with 5 mg/kg doxycycline or 10 mg/kg minocycline and 3/8 dogs treated with 5 mg/kg of minocycline were still positive for *Wolbachia* DNA at day 28. The potential impact of the remaining *Wolbachia* DNA on pulmonary pathology was not evaluated. The frequency and severity of gastrointestinal side effects (vomiting, diarrhea, weight loss) were greater in dogs receiving 10 mg/kg of either medication compared to 5 mg/kg.

Results are available at the 21 day time point for dogs treated with different doses of doxycycline, allowing for some comparison between dosages, albeit from different studies (79, 85). In 17 naturally infected dogs treated with doxycycline 10 mg/kg q 24 h for 21 days, *Wolbachia* DNA was detected in blood samples from 15/17 (88%) on treatment day 0, with a similar proportion positive at day 21 (14/17, 82%) (79). This decreased to 2/17 (12%) on day 111 (~16 weeks). When different tetracycline dosages were compared, at the 21 day time point 3/8 dogs (38%) treated with doxycycline 10 mg/kg q 12 h had *Wolbachia* DNA, compared with 6/8 of dogs (75%) treated with doxycycline 5 mg/kg q 12 h and 5/8 (63%) treated with either 5 or 10 mg/kg minocycline q 12 h (85).

*Wolbachia* positivity and microfilaremia rebounded 10 months after cessation of doxycycline in heartworm-positive dogs (79). One of the 7 dogs was positive 13 weeks after the end of the third treatment cycle; this increased to 4/5 dogs that were sampled 10 months after the last treatment. Microfilaria counts had increased concomitantly and were not significantly different from day 0 at this time point.

A third report demonstrated significant decreases in anti-*Wolbachia* IgG antibodies in 49 naturally infected dogs treated with doxycycline at 5 mg/kg q 12 h, 10 mg/kg q 12 h, or 10 mg/kg q 24 h for 30 days; none of these dogs showed evidence of clinical illness after adulticide therapy (86).

Finally, although commonly reported anecdotally, shorter durations of doxycycline treatment have not been investigated to date and could be of value, given the cost of the treatment and supply disruptions in recent years. As there has been no RCT focusing on clinical outcomes, it is not known which dose and duration of doxycycline (or minocycline) offer the greatest *clinical* advantage to the patient. The increased cost and potential for adverse effects of higher doses raise concerns about owner compliance and drug absorption (in the face of gastrointestinal upset). In animals who have adverse effects at the higher dosage, it is a common clinical practice to decrease the dose to finish the full duration of doxycycline therapy.

A 28- or 30-day course of doxycycline has demonstrable effects on treatment outcome and either 10 or 20 mg/kg/day is acceptable. For dogs that remain antigen positive 12 months after completing a course of doxycycline, the course should be repeated (2, 80); there are no known clinical benefits to repeating the treatment prior to that time point.

Non-arsenical therapy should not be initiated without adjunctive doxycycline.

## Moxi-Doxy: Testing to Confirm Treatment Efficacy

Treatment efficacy is considered to mean eradication of adult heartworms, which is clearly the ideal treatment endpoint. In endemic areas, however, particularly those where veterinary care is sporadic, inaccessible, or unaffordable, resolution of clinical signs (where present) should also be considered a valid treatment goal. While highly undesirable, reinfection is highly likely in many settings, and the resolution of clinical disease addresses immediate patient welfare concerns. While a discussion of immunity to complex multicellular parasites is well beyond the scope of this review, complex host immune responses occur (87, 88) and should not be entirely overlooked.

**Figure 2:**
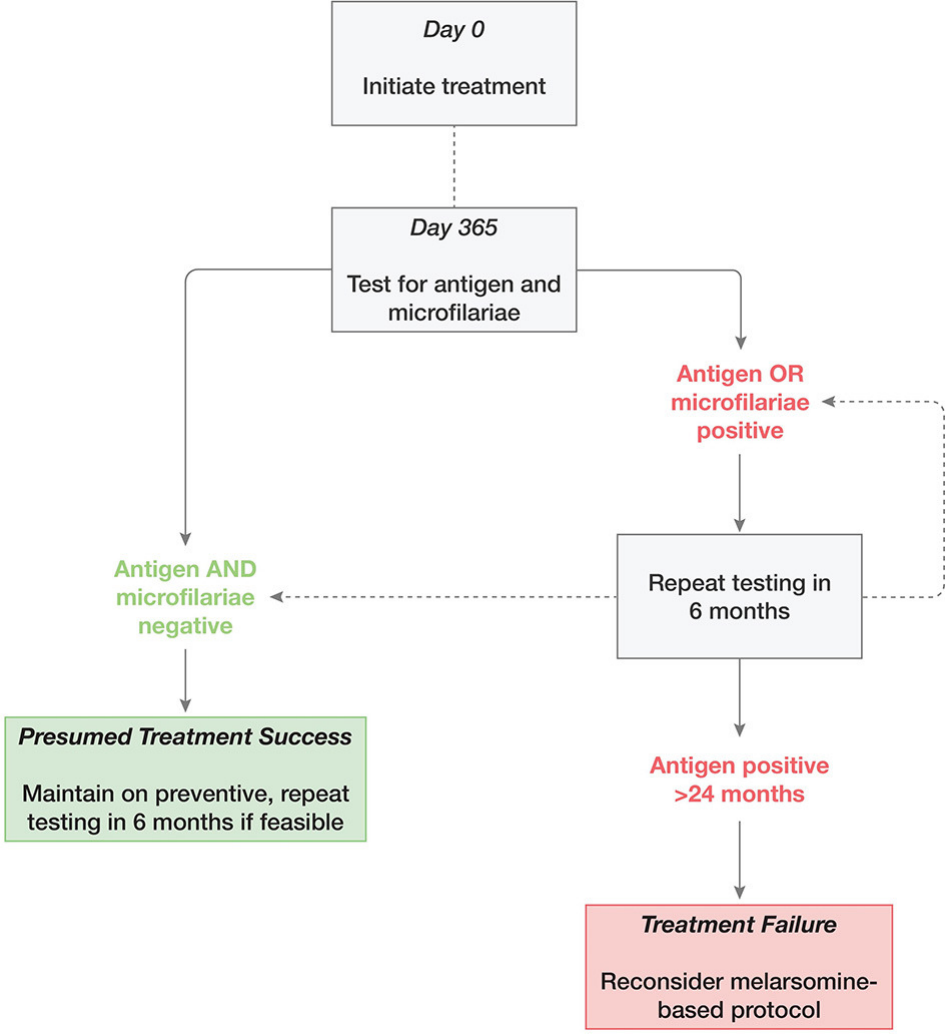
Suggested approach to retesting following adulticide treatment of heartworm infection with moxidectin and doxycycline.

The preferred treatment goal is NAD status—the best proxy for parasite elimination in clinical patients—together with a negative microfilarial test (Figure 2). A single negative antigen test is recommended to confirm efficacy after melarsomine treatment, with the caveat that a negative test does not rule out the presence of larval or juvenile stages, or a small number of adult male worms (2, 89). Determining NAD status is more complex for ML-doxycycline treated animals. The issues center around:

- Whether a conventional antigen test is likely to be a false negative; and
- Whether a single negative antigen test is adequate to discontinue treatment.

### Is Heat Treatment of Test Samples Required?

False negative antigen test results can be caused by antigen-blocking immune complexes (90). Immune complex dissociation (ICD), typically using heat, unmasks antigen that is blocked by antigen-antibody complexes. Heat treatment (HT) has demonstrated increased sensitivity in detecting early and male-only infections (89), but may also unmask residual antigen from dead and dying parasites (90). Results of the conventional, non-heat treated (NHT) antigen test and HT samples were compared in sera from shelter dogs, using necropsy counts of adult heartworms as the reference standard (89). Heat treatment increased the overall antigen test sensitivity from 87 to 95% (fewer false negatives), while also reducing specificity from 98 to 96% (more false positives). Heat treatment of sera from shelter dogs during screening for HWI modestly increased the proportion of positive results (91).

Previous treatment with MLs may exacerbate antigen blocking. Recent administration of a heartworm preventive was a significant risk factor for false negative results prior to HT (91). Heat treatment of sera after treatment of dogs with various ML-doxycycline combinations resulted in a high percentage of positive antigen tests after negative NHT results (92). In this small study, almost half of the dogs were inconsistently treated, with various protocols.

Dogs treated with either melarsomine or moxi-doxy had frequent conversions (>65%) from negative NHT to positive HT results (9), i.e., this phenomenon is not restricted to dogs treated with moxi-doxy. Three additional studies have compared HT and NHT antigen test results after moxi-doxy (48, 49, 93). In an experimental study, HT samples had higher optical density on the antigen test compared with NHT samples, even when both were positive (93). In a clinical study using samples taken every 6 months, HT samples initially disagreed with negative NHT samples in some dogs, with this discrepancy decreasing over time and no discrepancies at 18 and 24 months (49). All dogs that were NHT-negative/HT-positive were negative on both tests at the subsequent test 6 months later (49). In another clinical study, samples that were initially NHT-negative/HT-positive were all HT-negative 2–3 months later (48).

While HT is more sensitive for detection of residual HW antigen, this antigen may be released during and after HW death (90, 93) and the addition of this modification to the antigen test did not confer any diagnostic benefit following adulticide treatment with moxi-doxy (9, 48, 49, 93). Heat treatment is therefore not recommended when determining NAD status during moxi-doxy treatment.

### Timing of Retesting

Based on findings to date (Table 1), approximately half of dogs may have NAD as early as 6 months after initiation of moxi-doxy treatment, with ~90% NAD by 12 months. Conversion to NAD status is expected to be slower in dogs with higher worm burdens (61). In one study, middle-aged dogs had higher worm burdens than younger and older dogs (94). Microfilaria count is not a reliable proxy for worm burden (94); however, NAD status was achieved earlier in dogs with lower microfilaria counts (49).

In general, retesting can reasonably be considered 6 months after initiation of treatment, but later testing (at 12 months) is a more appropriate use of limited resources (Figure 2). In addition to the severity of clinical signs, microfilaremia and age, timing should be based on practical considerations such as local experience, owner preference and resources, frequency of access to veterinary care, and adoption considerations (for shelters and rescues).

### Should Negative Antigen Tests Be Repeated to Confirm NAD Status?

Two negative tests, 6 months apart, have been recommended to confirm NAD status after moxi-doxy treatment (2, 49). One study reported some variation in antigen test results and therefore defined NAD status as a minimum of two negative test results (9). Dogs were tested frequently in this study, initially monthly and then every 3 months from 9 to 18 months, compared with every 6 months in most other studies. There is no specific evidence that two tests are required at 6-month intervals. In studies where testing occurred at this frequency, no case was reported where a dog tested NAD at one time point and then positive at a subsequent time point (48, 49, 52).

The advantage of a second test is the reassurance of 6 months of additional treatment in the interim and the assurance that NAD status has persisted. Further, it would demonstrate that all stages of the life cycle have been accounted for. However, repeat testing and additional treatment may be impractical in remote communities or where other barriers to care exist.

## Safety, Welfare, and Population Concerns

A number of safety, welfare, and population concerns have been raised regarding moxi-doxy treatment. These are addressed below and summarized in Table 4.

**Table 4:** Safety, welfare, and population concerns regarding heartworm adulticide treatment in dogs, using moxidectin and doxycycline.

**Concern**	**Rationale**	**Assessment**
Pulmonary pathology	•Continued inflammatory response to the heartworms during prolonged death	•Lesser harm than leaving heartworms untreated •Mitigated by doxycycline •AHS 3-dose melarsomine protocol delays melarsomine until 60 days post-diagnosis (however some published evidence supports initiating melarsomine at 30 days post-diagnosis)
Exercise restriction	•Long period of exercise restriction until no antigen detected status is reached •Burden on owner, welfare concern for dogs	•Serious complications rare even without strict exercise restriction •More studies are needed to determine the optimal duration and extent of exercise restriction for moxi-doxy vs. melarsomine
Compliance with monthly medication	•Long duration of treatment •Treatment may be stopped by owner due to financial considerations, clinical improvement, limited access to medication	•Similar issues for preventives, which are also needed for these dogs post-treatment •Sustained release formulations offer a solution; more data needed for efficacy and optimal dosage regimen
Maintenance of a reservoir of infection	•Adults remain alive for extended period	•Lesser harm than leaving heartworms untreated •Moxidectin quickly clears microfilariae, breaking transmission cycle •Treatment may also interrupt transmission before death of adult worms through effects on embryogenesis and larval viability
Selection for microfilarial resistance	•Long duration of low concentrations of moxidectin may select for resistance	•Evidence suggests that moxidectin may be effective against resistant microfilariae •This concern also exists with year-round preventives, given known compliance issues
Ultimate cost savings may not be significant	•Long duration of treatment may cancel the cost savings of the non-arsenical protocol •Long length of stay and time in care is also a significant concern for shelters and rescues	•Offering a spectrum of care approach and payment plans can reduce costs and make melarsomine more accessible •Clear communication about total costs, including clinic visits, medication and testing, is required •Barrier may be up-front cost, not total cost •Melarsomine may be refused for reasons other than financial

### Progression of Pulmonary Pathology

A primary objection to non-arsenical protocols, as regards patient safety and welfare, is that live adult worms remain in the pulmonary arteries for a prolonged period. This could result in progressive pulmonary endarteritis, pulmonary hypertension, perivascular inflammation, and potentially right heart failure (2). It is important to note that in the patient populations discussed here, the decision is not a choice between melarsomine and ML-doxycycline, but between a non-arsenical protocol and no treatment at all.

Measures of the extent and progression of pulmonary damage are difficult to assess accurately in clinical studies and without advanced imaging; this is further complicated by the fact that changes are not always reversible following treatment (95, 96).

When dogs with experimental adult HWI were treated with IVM, there was no difference in the progression of pulmonary disease (arterial and interstitial disease and pulmonary hypertension) when compared with milbemycin or no treatment (97). Both MLs were given according to label recommendations. When treatment was begun while the heartworms were immature, interstitial lung disease and increases in pulmonary arterial diameter were more severe in the IVM group at 9–11 months post-infection than in the milbemycin group and resembled changes in control dogs. Scores for IVM and milbemycin were similar beyond this time point. In a study of owned heartworm-positive dogs that were treated with monthly IVM for 24 months, 11/14 treated dogs (79%) and 3/3 untreated controls had normal echocardiographic and radiographic scores, that remained normal at the end of the treatment period (98). Two of 11 dogs treated with IVM (18%) had normal scores initially and developed abnormalities over the treatment period, and one had abnormal scores that worsened.

Both ML and doxycycline treatment are recommended prior to melarsomine, to reduce the worm burden and eliminate pro-inflammatory antigens, thereby reducing post-adulticide complications (33, 82). Doxycycline reduced the pulmonary inflammatory response to dead and dying worms when given prior to melarsomine, with a dramatically greater effect when IVM was added (82). This ameliorating effect could help offset the negative impact of slower worm death in ML-doxycycline protocols. MLs and doxycycline work synergistically to damage and eliminate pre-adult stages of *D. immitis* (83, 84). This prevents the development of additional adult worms during ML-doxycycline treatment and in this way may also help to slow disease progression, as compared with no treatment at all. This advantage may, however, be offset by the inflammatory response to dead and dying worms during treatment.

Clinical signs were not detected in dogs despite documented pulmonary changes during treatment with IVM (97). An asymptomatic presentation cannot be assumed to mean that pulmonary pathology is absent (96). However, in practical terms, clinical signs are of most concern to the owner and have the greatest welfare implications for the dog.

Five moxi-doxy studies have reported clinical, radiographic and/or echocardiographic findings (Table 5). New or worsening respiratory signs during moxi-doxy treatment have been absent or mild in the majority of dogs (9, 31, 48, 55) (Table 5), with only one dog in these studies requiring brief hospitalization (48). When moxi-doxy was compared with 2-dose melarsomine (without doxycycline), respiratory complications occurred in 4/15 dogs in the moxi-doxy group (27%) and 2/15 in the melarsomine group (13%) (9). Three of the 4 affected dogs in the moxi-doxy group required prednisone at ~2, 3, and 5 months, respectively. Another dog had parasitic pneumonitis and required intermittent prednisone (9). Hemoptysis and tachypnea were seen in the melarsomine group. In dogs treated with melarsomine, respiratory complications (including 2 deaths) decreased from 9/47 (19%) without doxycycline to 3/47 (7%) with doxycycline (81).

**Table 5A:** Cardiopulmonary changes during heartworm adulticide treatment in dogs, using moxidectin and doxycycline.

**References**	**Study protocol**	**Exercise restriction**	**Summary findings**
Savadelis et al. (31), Savadelis et al. (53)	EI M/I + doxy (*n* = 8)	Moderate; housed in runs, in pairs	•Cough, quiet demeanor and crackles in 1/8 at ~1 month – treatment not needed •Suspected PTE in 1 infected dog (13%) at d 173 (6 m); resolved spontaneously within 24 h •Radiographic and echo evidence of disease similar to untreated controls •Arterial thrombi scores significantly higher in treated dogs, attributed to dead and dying worms
Paterson et al. (9)	NI M/I + doxy (*n* = 15)	Recommended, but many were free-roaming	•4/15 (27%) developed cough requiring treatment •3 required prednisone on only one occasion; 1 required prednisone intermittently for parasitic pneumonitis that was present at enrollment •Onset of new cough occurred 3 to 5 months after initiation of treatment
Genchi et al. (52)	NI M/I + doxy (*n* = 14)	Not stated	Interstitial lung scores on radiographs, worst possible score 3/3. Echo scores based on pulmonary hypertension, worst possible score 3/3 •No dog had radiographic or echo score > 1/3 at enrollment •Radiographic score progressed in 1/14 (7%) at 6 months (from 0/3 to 1/3) •All radiographic scores normal (0/3) from 12 months •Two dogs (14%) abnormal echo scores (1/3) at enrollment •All other echo scores normal at all time points
Ames et al. (48)	NI M/I + doxy (*n* = 22)	None	•5/22 (23%) – cough at enrollment; no treatment needed; worsened in 2/5 during treatment •8/22 (36%) – new or worse cough during treatment, onset 60–180 (mean 89 d); 4 required treatment •1/22 (5%) – dyspnea and cough at 12 w, pneumonitis, hospitalized and recovered •Subjective radiographic severity scores, with worst possible score 3/3 – baseline median 1; peak 1.25 •Peak score occurred at an average of 58 d after initiating treatment •Highest scores (3/3) in two dogs with cough requiring treatment, on days 60 and 84 •Nine (56%) had normal (0/3) to mild scores (1/3).
Alberigi et al. (55)	NI PH + doxy (*n* = 20)	Mild to moderate	•Statistically significant improvement in most measures (cough, dyspnea, expiratory sounds, pulmonary artery enlargement) by time of first NAD, several measures improved at second NAD •Bronchointerstitial pattern in all dogs at intake; no significant improvement •Micronodular pattern prevalence increased at time of first NAD, returned to baseline at second NAD (7/19 vs. 6/19 at intake)

*NI, natural infection; EI, experimental infection; M/I, moxidectin/imidacloprid as Advantage Multi® or Advocate® PH, moxidectin as ProHeart® 12; echo, echocardiogram/echocardiographic; PTE, pulmonary thromboembolism; NAD, No antigen detected*.

**Table 5B:** Cardiopulmonary changes during heartworm adulticide treatment in dogs, for control groups from studies of moxidectin and doxycycline.

**References**	**Study protocol**	**Exercise restriction**	**Summary findings**
Savadelis et al. (31, 53)	EI Untreated controls (*n* = 8)	Moderate; housed in runs, in pairs	•Cough in 1/8
Paterson et al. (9)	NI 2-dose melarsomine without doxy (*n* = 15)	Recommended, but many were free-roaming	•One developed hemoptysis on day 9 and required prednisone •One developed tachypnea after the first melarsomine injection
Genchi et al. (52)	NI 3-dose melarsomine without doxy (*n* = 6)	Not stated	Interstitial lung scores on radiographs, worst possible score 3/3. Echo scores based on pulmonary hypertension, worst possible score 3/3 •Two dogs (33%) progressed radiographically from 0/3 to 1/3 at 12 months •All radiographic scores normal at 12 and 18 months •Two had pulmonary hypertension (1/3) at enrollment and 6 months; 1 developed mild new pulmonary hypertension at 6 months (0.5/3) •All other echo scores normal at all time points

*NI, natural infection; EI, experimental infection; echo, echocardiogram/echocardiographic*.

The current AHS guidelines recommend prednisone as a routine adjunctive treatment to help reduce the risks of clinical pulmonary thromboembolism (2). This further complicates efforts to compare rates for respiratory signs and complications between arsenical and non-arsenical protocols, as studies evaluating moxi-doxy protocols have not included the routine use of glucocorticoids. While prednisone may not be required for all dogs receiving the 3-dose melarsomine protocol (99), the added anti-inflammatory effect may be of benefit as part of moxi-doxy protocols. Timing and duration are likely to be extremely difficult to ascertain, however, because of the unpredictable timing of worm death.

In an RCT involving experimentally infected dogs, clinical and necropsy findings were compared between those treated with moxi-doxy and untreated controls over 10 months (31) (Table 5). Radiographic abnormalities were not significantly different between the groups at any time point, but an interstitial or alveolar pattern score was higher than the baseline score on more study days for the treatment group. Echocardiographic scores did not differ between the groups. The histopathological arterial thrombus score was significantly higher for the treated group, an expected finding following worm death with any adulticidal treatment (31). The differences identified in this study were considered to have minimal or indeterminate clinical relevance during the time period examined (31); however, the study demonstrated that pulmonary changes did not improve during the early stages of moxi-doxy treatment.

In naturally infected dogs treated with moxi-doxy (*n* = 14) or 3 doses of melarsomine (without doxycycline) (*n* = 6), the majority of dogs in both groups had normal radiographic scores at all time points up to 24 months (52) (Table 5). One dog in the moxi-doxy group and 2 in the melarsomine group had mild new radiographic changes at 6 or 12 months, and all returned to normal 6 months later. Moxi-doxy was non-inferior to melarsomine for cardiac ultrasound scores, which were abnormal at baseline in 2 dogs in each group, became mildly abnormal at 6 months in one dog in the melarsomine group, and were normal in all dogs from the 12 month time point. In this study, therefore, there was mild, temporary progression of cardiac or pulmonary imaging scores in a small number of dogs, with the majority remaining normal throughout and the remainder improving over time. Similar trends were seen for dogs treated with IVM-doxy (100).

In 16 naturally-infected dogs treated with moxi-doxy that had radiographs taken, subjective grading showed mild average scores at enrollment (mean 1.04, median 1 of a possible worst score of 3), with slight worsening at 2 months (mean 1.44, median 1.25) (48) (Table 5). Nine of 16 dogs had normal to mildly abnormal radiographs. A dog that developed severe clinical signs was scored at 3/3 after 84 days of treatment and another, that developed a cough, had a score of 3/3 at 60 days.

Eight dogs had pulmonary arterial (PA) enlargement at the initiation of treatment with SR injectable moxidectin and doxycycline (55). This had resolved in several dogs (3/8 for caudal PA enlargement and 4/8 for main PA enlargement) at the first negative antigen test and in the majority (6/8 and 5/8, respectively) at the second negative antigen test. Bronchial and interstitial patterns were present in 19 dogs at enrollment and only resolved in 1/19. Micronodular pulmonary patterns became more common during treatment, being present in 6 dogs at enrollment, 9 at the first negative antigen test and 7 at the second negative antigen test.

Biomarkers have been used as a measure of the severity of cardiopulmonary changes in HWD (61, 101–104). These have not been measured during moxi-doxy treatment but were assessed in dogs given IVM-doxy (61). Concentrations of biomarkers decreased significantly by 10 months in all dogs treated with IVM-doxy but, in some dogs, some biomarkers remained elevated at the end of treatment (6 months) and/or at the end of the study. This was more frequently the case for dogs with Class 3 HWD (61). In both studies, dogs treated with melarsomine showed significant reductions in biomarkers over the course of treatment, with improvements considered to be markedly better than for IVM-doxy (a direct comparison was not performed) (61, 104). These findings support other evidence that less cardiopulmonary damage occurs in melarsomine-based treatment protocols.

Current AHS Guidelines recommend delaying treatment with melarsomine for 60 days after diagnosis of HWI (2), during which period MLs and doxycycline are administered. The reason for this delay was to overcome the so-called “susceptibility gap,” a period during which some stages of the parasite would not respond to either ML or adulticide treatment (105). However, this gap may not exist when 2- or 3-dose melarsomine protocols are used in conjunction with MLs, resulting in a recommendation to begin melarsomine as soon as possible after diagnosis (105).

A 60-day delay in melarsomine treatment also allows *Wolbachia* proteins and metabolites to be eliminated and weakens adult worms (2). During this period, the progression of pulmonary disease would be identical in the conventional and non-arsenical protocols. Parasite death is, however, much more rapid after initiation of melarsomine, with the elimination of adults within 2–3 months (without doxycycline) (Table 3) (6, 106).

In summary, the majority of dogs treated with moxi-doxy have shown no to mild respiratory complications, with only a few dogs requiring treatment and one needing brief hospitalization; no HW-associated deaths were reported (9, 31, 48, 52, 53, 55). This is despite the lack of strict exercise restriction in most cases. To date, there have been no RCTs directly comparing respiratory complications for 3-dose melarsomine protocols and moxi-doxy. Since clinical repiratory complications are rare and generally mild, the possibility of progression of pulmonary damage should not be a reason to withhold moxi-doxy from dogs with HWI when melarsomine is not a viable treatment option.

### Exercise Restriction

Exercise restriction is recommended from the time of diagnosis of HWI until 6–8 weeks after the third melarsomine treatment (2) (i.e., ~5 months in total, including the 3-month higher risk period during and after rapid worm death). This is essential to help prevent cardiorespiratory complications (2).

The required stringency and duration of exercise restriction are unknown for ML-doxycycline protocols. In rural and remote communities in particular, many dogs roam outdoors and strict exercise restriction may be impracticable or require tethering. A prolonged period of exercise restriction without enrichment raises welfare concerns for dogs and may lead to behavioral problems that have implications for owners and the human-animal bond.

Of 79 dogs treated with moxi-doxy and not subjected to strict exercise restriction, the majority had an uncomplicated treatment course (9, 31, 48, 52, 55), with mild respiratory complications in some dogs (new or worsening cough) (Table 5). There were more serious, but short-lived, complications in two dogs (31, 48) and parasitic pneumonitis requiring intermittent treatment in one (9). No deaths were reported due to complications and only one dog required hospitalization for 24 h.

The potential for serious complications in some dogs nonetheless dictates that at least some degree of exercise restriction must be recommended (48), especially in the first several months of treatment. When treating HWI in any dog, the more severe the clinical signs, the more severe the exercise restriction should be (2). High worm burdens can be present in some dogs without proliferative lesions in the pulmonary arteries (107). Such dogs might not show clinical signs but could still be at high risk for pulmonary thromboembolism. A discussion of risk-benefit and the feasibility of strict exercise restriction should be held when dogs are treated with moxi-doxy; some level of clinician and owner discretion is required.

Prolonged length of stay in an animal shelter setting is a documented threat to both physical and behavioral health and welfare and must be avoided (108). Holding an animal in a shelter until they achieve NAD status is unacceptable and the capacity to provide for the needs of heartworm positive animals should be assessed prior to initiating treatment. In one survey, the majority of animal shelters treating heartworm positive dogs did so through the use of foster or foster-to-adopt programs (5). Regardless of the treatment protocol and operational programming in place, arrangements should be made for follow-up care and monitoring after adoption until such care can be transitioned to the new veterinarian.

### Selection for Microfilarial Resistance

Resistance to HW preventives remains a significant concern (109–111), and concern about selection pressure for microfilarial resistance has been an important objection to non-arsenical treatment protocols. Small geographic areas of resistance have been documented in high-prevalence regions of the US (112). Resistance does not, however, appear to be common nor increasing in prevalence, with apparent lack of efficacy explained by other factors in most cases (73, 113). Identification and characterization of resistant isolates is challenging (112). It is unclear whether year-round prevention, as recommended for many regions, would necessarily result in less selection pressure than ML-doxycycline treatment of infected dogs.

Moxidectin has been reported to be more effective against resistant microfilariae than other MLs (62, 67, 114). This is thought to be due to its physicochemical and pharmacokinetic properties, in particular its high potency, distribution to fat and redistribution to plasma, resulting in longer persistence in the host and longer duration of action (62, 67).

The addition of doxycycline to treatment protocols has further reduced the risk of creating ML resistance, by interrupting embryogenesis and larval transmission (56, 58, 80, 115). Moxidectin is also directly microfilaricidal at the topical dose (69). Microfilariae are rapidly eliminated by moxi-doxy, frequently within 3–4 weeks (48, 49, 52, 53, 55). Elimination was more rapid than for IVM-doxycycline (56, 57), although direct comparisons are not available.

Based on these factors, the risk of selection for resistance might be lower for moxi-doxy than for previous ML treatment protocols, but more evidence is needed. Importantly, rapid clearance of microfilaremia in most cases (given that resistance remains uncommon) means that infected dogs treated with moxi-doxy will no longer be a reservoir of infection for at-risk animals in the community.

## Conclusion

There is now an adequate body of evidence to demonstrate that moxi-doxy protocols are a viable option for adulticide treatment of HWI in dogs for which melarsomine treatment is not an option. In cases in which melarsomine is available, but the 3-dose protocol is not feasible for an owner or shelter, a modified melarsomine protocol (2 doses, beginning after a month of doxycycline) should first be considered before non-arsenical treatment is recommended. If non-arsenical adulticide treatment is the only viable treatment option, moxi-doxy protocols are preferred to other non-arsenical protocols. There is now adequate data to support recommendations for more uniform treatment and post-treatment testing protocols. Evidence-based recommendations for the use of moxi-doxy as an adulticide treatment are provided in Table 6.

**Table 6:** Recommendations for moxidectin-doxycycline adulticidal treatment of asymptomatic and mild (Class 1 and 2) heartworm disease in dogs.

**Element**	**Recommendation**
General	•Not recommended in best practice guidelines •Viable treatment alternative if melarsomine is not a treatment option •Outcomes are likely to be less favorable and treatment duration longer for Class 3 disease, which is likely to represent higher worm burdens and more chronic infection
Moxidectin	•Topical formulation recommended •Administer at label dose, monthly until NAD •Consider sustained release injectable in remote communities or where significant compliance or accessibility concerns exist •Unknown whether sustained release formulation should be used per label or more frequently •No data to date for oral formulation
Doxycycline	•Essential component of any adulticidal treatment protocol •10 mg/kg PO q 12 or 24 h for 28 days •Repeat annually if still positive
Duration of treatment	•Continue until NAD
Exercise restriction	•Clinical PTE appears to be rare but timing is unpredictable •Insufficient data to be confident of required duration or stringency •Restrict exercise to the extent possible until NAD, commensurate with clinical severity, owner's ability to implement and dog's lifestyle and temperament
Testing for efficacy (NAD)	•Antigen testing at 6 months – cost:benefit analysis needed as approximately half are expected to remain positive at this point •Antigen testing at 12 months likely to give a negative result in the majority of cases •Test for microfilaremia at time of antigen testing to identify resistant parasites •Unclear whether 2 antigen tests, 6 months apart, are needed – current recommendation is to test twice where possible, as a precaution •No evidence that heat treatment is required to determine NAD status

*NAD, no antigen detected; PTE, pulmonary thromboembolism*.

There is a need for RCTs that address several important concerns about moxi-doxy. For example, no RCT has compared respiratory complications in the same population of dogs treated with the currently recommended 3-dose melarsomine protocol and moxi-doxy, and no RCT has compared clinical outcomes for different degrees and durations of exercise restriction in dogs treated with moxi-doxy. Only one study has been published for SR injectable moxidectin as an adulticide treatment, and none for oral moxidectin. Such studies would be valuable additions to the literature.

Veterinarians using the “least harm” principle to expand treatment options for heartworm positive dogs should have a thorough understanding of the risks and benefits of non-arsenical treatment protocols, including moxi-doxy. Despite its limitations, the authors believe that the current evidence demonstrates that the benefits of moxi-doxy for affected individuals and at-risk populations outweigh the risks of untreated HWI. Moxi-doxy protocols can provide lifesaving relief for dogs and communities that lack access to melarsomine or routine veterinary care.

## Author Contributions

All authors have made a substantial, direct and intellectual contribution to the work, and approved the final version.

## Conflict of Interest

The authors declare that the research was conducted in the absence of any commercial or financial relationships that could be construed as a potential conflict of interest.

## Publisher's Note

All claims expressed in this article are solely those of the authors and do not necessarily represent those of their affiliated organizations, or those of the publisher, the editors and the reviewers. Any product that may be evaluated in this article, or claim that may be made by its manufacturer, is not guaranteed or endorsed by the publisher.

## Footnotes

^1^https://www.avma.org/javma-news/2004-10-15/fort-dodge-recalls-proheart-6-citing-fda-safety-concerns.
